# Relationship between learning flow and academic performance among students: a systematic evaluation and meta-analysis

**DOI:** 10.3389/fpsyg.2023.1270642

**Published:** 2023-11-06

**Authors:** Zhang Jinmin, Fang Qi

**Affiliations:** ^1^School of Physical Education and Sport Science, Fujian Normal University, Fuzhou, China; ^2^Chengdu Sport University, Chengdu, China

**Keywords:** learning flow, academic performance, systematic evaluation, meta-analysis, relationship

## Abstract

**Introduction:**

The concept of “flow experience,” characterized by a state of immersive enjoyment and profound engagement, pertains to individuals’ deep involvement in intriguing and pleasant tasks. In the field of study, individuals are in a state of flow when encountering challenging tasks, which matters considerably in completing the tasks. Therefore, learning flow is considered a hotspot in education that may be related to improving academic performance. Nonetheless, there remains contention regarding the extent of learning flow’s impact on academic performance. To this end, meta-learning was hereby used to provide evidenced on the relationship between them.

**Methods:**

A systematic review was conducted under the guidance of PRISMA to examine the evidence of learning flow and academic performance, check the potential mechanism and evaluate the current evidence. Clinical research or empirical research on the influence of learning flow on academic achievement was collected by searching four databases. The literature retrieval spanned from each database’s inception until June 2023, specifically covering the PubMed (2000–2023.6), Embase (1974–2023.6), Cochrane Library (1993–2023.6), and the Web of Science (1807–2023.6), with particular attention to the period between 2000 and 2023.

**Results:**

Thirteen RCTs were included, the total sample size used in the study was 3,253. Using the NOS evaluation tool of queue study, the average evaluation score of the included literatures was 7.46, indicating that the overall literature was above average. Besides, the data software StataSE was used to test the heterogeneity of the data, and the correlation coefficient and 95% confidence interval effect were found to be 0.43 (0.28, 0.57).

**Discussion:**

Our research indicates a link between learning flow and academic performance, that is, students with high learning flow levels tend to have better academic performance. At the same time, this conclusion needs to be verified by more high-quality literature and larger sample data.

**Systematic review registration:**

https://inplasy.com, identifier INPLASY202360079.

## 1. Introduction

“Flow” reflects a state of profound concentration or complete absorption in an activity (Kaya and Ercag, 2023). This concept encompasses nine critical dimensions: challenge-skill balance, immediate and lucid feedback, unequivocal goals, deep concentration, harmonious action, heightened self-awareness, a robust sense of control, altered perception of time, and engagement in the task at hand. The initial trio serves as the foundational prerequisites for flow, while the subsequent sextet characterizes its intrinsic state. Notably, this phenomenon permeates academic endeavors (Oertig et al., 2014), rendering “learning flow” a descriptor for students’ optimal engagement, wherein they relish the learning process, devoid of ennui or angst (Huang and Wang, 2022). In this milieu, students attain a flow state when challenges align with their skillsets, bolstered by explicit objectives and prompt, constructive feedback. This harmonious state fosters profound self-awareness and self-regulation, culminating in immersive task execution and a distorted sense of time, typically engendering positive educational outcomes. Consequently, learning flow is pivotal in educational engagement, cognitive evolution, self-efficacy, and academic performance (Kim and Lee, 2021).

For students, academic performance reflects personal past performance results, and also provides key information concerning students’ mastery of academic activities and technical skills, mattering considerably in their participating in academic related tasks and achieving success in educational activities (Brown et al., 2016). For children, academic performance is usually assessed by transcripts or standardized national tests (Smith et al., 2023). For example, the so-called CITO tests (made by the Central Institute for Tests Development in the Netherlands) is used to monitor the learning progress of Dutch primary school children in mathematics, reading, spelling and reading comprehension from grade one to grade six (Van Tetering et al., 2018). The core of Germany’s SASCHA (Social and Academic School transition CHAllenges) is to study the social and academic challenges faced by children when transitioning from the primary school to the secondary school, and to assess their average scores in mathematics, German and English (Blume et al., 2022). In Chinese schools, students’ performance in subjects of Chinese, mathematics and English are usually used for the evaluation of their academic achievements. For college students, GPA is considered a good predictor of their academic performance (Kaya and Ercag, 2023). Current research indicates that primary influences on academic performance divide into external and internal factors. External elements comprise course content, subject expertise, and social interaction, encompassing teaching methods and peer engagement. Conversely, internal elements primarily involve learners’ personal competencies, cognitive styles, psychological characteristics, and self-efficacy, among others (Liu et al., 2022). Within this context, ‘learning flow’ emerges as a potential determinant of academic outcomes, potentially impacting knowledge structures, social dynamics, and personal characteristics.

It is important to examine the relationship between learning flow and academic achievement. The impact of flow experience on school performance was analyzed. A total of 697 eighth grade students with an average age of 13.4 years completed a questionnaire that included the measurement of self-control, school performance and learning flow at the beginning and end of the school year. The study revealed a correlation coefficient of 0.14, demonstrating that learning flow was significantly correlated with academic performance (Kuhnle et al., 2012). To find the moderating effect of learning flow and academic achievement in online learning, a study on 272 nursing students from six universities in two different cities was conducted using self-reported questionnaires to measure learning flow, learning process, digital literacy and academic achievements. The study found that the mediating effect of learning flow on academic performance was 0.42, with a stronger learning flow indicating a stronger academic performance, which was confirmed to further improve academic performance (Ryu et al., 2022). However, randomized experimental research was designed to explore how to improve students’ enthusiasm, flow and academic success through the generated competitions and challenges. A total of 30 college students in the control group and 30 in the experimental group used the educational application for one semester. The study found no significant difference in the level of flow experience between the control group (Yeh et al., 2019). To reveal the mindfulness learning experience, learning flow, self-efficacy and mastery experience, another study recruited 83 students from the fifth and sixth grade participating in a six-week game-based creativity learning program. The researchers found the path model analysis did not support the direct impact of flow experience on mastery experience mindfulness learning and self-efficacy (Adil et al., 2020). To conclude, the influence of learning flow on students’ academic achievements is not uniform, indicating an ongoing disparity in viewpoints concerning its potential effects. Therefore, Meta-analysis was hereby used to clarify the relationship between them. This study contributes considerably to students’ learning and education, and possesses better clinical significance for improving students’ academic performance. Meanwhile, this paper also facilitates to promote flow learning strategy. The purpose of this study is to explore the relationship between learning flow and academic performance. Therefore, the central question of this study is whether there’s a relationship between students’ learning flow and their academic performance.

## 2. Methods

### 2.1. Agreement

The review procedure follows the statement of the Preferred Reporting Item (PRISMA) for systematic review and meta-analysis (Moher et al., 2009). The PRISMA list can be provided as a Supplementary document, and the systematic review, search strategies, meta-analysis and appendix detailed can be obtained from the Open Science Framework (https://osf.io/cqmjb/).

### 2.2. Retrieval strategy

Four electronic databases (Pubmed, EMBASE, Cochrane Central Register of Controlled Trials and Web of Science) from the creation to June 2023 were searched. Literature from 2000 to 2023 was included. And the detailed retrieval strategy is shown in Table 1 (taking Pubmed as an example).

**Table 1:** Search strategy on PubMed.

Search	Query	Results
#1	“learning flow” [All Fields]	10,584
#2	((((((((((((((((((((((((achievement[MeSH Major Topic]) OR (Achievements[Title/Abstract])) OR (Accomplishment[Title/Abstract])) OR (Accomplishments[Title/Abstract])) OR (Academic Successes[Title/Abstract])) OR (Success, Academic[Title/Abstract])) OR (Successes, Academic[Title/Abstract])) OR (Academic Achievement[Title/Abstract])) OR (Academic Achievements[Title/Abstract])) OR (Achievement, Academic[Title/Abstract])) OR (Achievements, Academic[Title/Abstract])) OR (Achievement, Educational[Title/Abstract])) OR (Educational Achievements[Title/Abstract])) OR (Educational Level[Title/Abstract])) OR (Educational Levels[Title/Abstract])) OR (Level, Educational[Title/Abstract])) OR (Status, Educational[Title/Abstract])) OR (Education Level[Title/Abstract])) OR (Education Levels[Title/Abstract])) OR (Level, Education[Title/Abstract])) OR (Level of Education[Title/Abstract])) OR (Educational Attainment[Title/Abstract])) OR (Attainment, Educational[Title/Abstract])) OR (Educational Attainments[Title/Abstract])) OR (Educational Achievement[Title/Abstract])	132,802
#3	#1 AND #2	72

### 2.3. Inclusion criteria

(1) The experimental group was used to enhance the learning flow; (2) only routine learning was conducted for students in the control group. Conventional learning refers to the teaching style; (3) clinical research or empirical research was carried out; and (4) the outcome indicators included at least one of the following: achievement test scores, mobile questionnaires, or scales. The former materializes through summative assessments or traditional paper examinations, appraising students’ academic performance. In contrast, in latter, instruments such as mobile questionnaires (Pearce et al., 2005), the Flow State Scale (Jackson and Marsh, 1996), or specifically designed scales for online learning environments utilize a Likert five-point format for assessment (Esteban-Millat et al., 2014). Quantitative data on the relationship between learning flow and academic achievement were included.

### 2.4. Exclusion criteria

(1) Incomplete data, unreported studies or animal studies; or (2) literature types such as programs, meeting summaries, reports or letters.

### 2.5. Screening process

Endnote was used to screen and exclude the literature. Firstly, the researchers read the title of the articles to delete the duplicate literature. Secondly, researchers read abstracts to judge whether the articles were related to the theme. Finally, the researchers read the full text to include it. Any research failing to meet the criteria would be excluded, and any differences would be resolved through discussions.

### 2.6. Literature quality assessment

A double check and then a quality evaluation were carried out, after which, Kappa test was conducted, and a high consistency was observed between the two people. The KPAP value was 0.87, indicating a high consistency. The quality evaluation scale was adapted from the quality index (Downs and Black, 1998), the research article evaluation list (DuRant, 1994) and the evaluation tool (Genaidy et al., 2007), with a total score of 9 points. Articles with a score of ≤ 6 points were defined as low quality literature; 7 ≤ the score ≤ 8, medium quality literature; and the score ≥ 9, high-quality literature. The details are shown in Table 2.

**Table 2:** NOS evaluation criteria for queue study.

Column	Entry#	Evaluation Criterion
Study population selection	How representative is the exposure group (1 point)	① It truly represents the characteristics of the exposure group in the population*; ② To some extent, it represents the characteristics of the exposure group in the population*; ③ Select a certain group of people, such as medical students; ④ Source of exposure group is not described
	Selection method of the non-exposure group (1 point)	① From the same population*; ② From different populations; ③ The source of the non-exposure group is not described
	Determination method of exposure factors (1 point)	① Fixed file records*; ② Structured interview*; ③ Reports written by the research subjects themselves; ④ Not described
	Determine the outcome indicators that do not need to be observed at the beginning of the study (1 point)	① Yes*; ② No
Comparability between groups	Consider the comparability of exposed group and non-exposed group when involving and statistical analysis (2 points)	① The study controlled the most important confounding factor*; ② The study controlled for any other confounding factors*
Result measurement	Whether the evaluation of research results is sufficient (1 point)	① Blind independent evaluation*; ② Archives*; ③ Self-reporting; ④ Not described
	Whether the follow-up time is enough after the occurrence of results (1 point)	① Yes (specify appropriate follow-up time before evaluation)*; ② No
	Whether the follow-up of exposure group and non-exposure group is sufficient (1 point)	① Follow up was complete*; ② A small number of subjects lost interviews without bias*; ③ Loss of follow-up but not described; ④ Follow-up is not described

# score entry.

* score points.

### 2.7. Data extraction

Data extraction tables were used to record the data in the study, including first author, country, year of publication, sample size (number of totals, men and women), average age (mean and standard deviation), intervention details and NOS (Newcastle-Ottawa Scale) score.

Herein, through preliminary synthesis of research results, themes and relationships were explored, and connected with research questions (Popay et al., 2006). Then, quantitative synthesis was chosen to examine the relationship between learning flow and academic performance in a series of tasks, environments and populations (Gurevitch et al., 2018). Since most studies reported correlation analysis, the meta-analysis adopted the R value. The “metafur” of R was used to calculate the size of the combined effect (Viechtbauer, 2010). Given that the size of the effect used in quantitative synthesis was not derived from the homogeneous population, the random effect model was selected to better explain the statistical heterogeneity in the study (Borenstein et al., 2009). To assess the robustness of synthesis results, we conducted a “leave-one-out” analysis. To identify potential biases in smaller studies, we generated a funnel plot, which was visually inspected for symmetry following established criteria.

## 3. Results

### 3.1. Study identification and selection process

**Figure 1:**
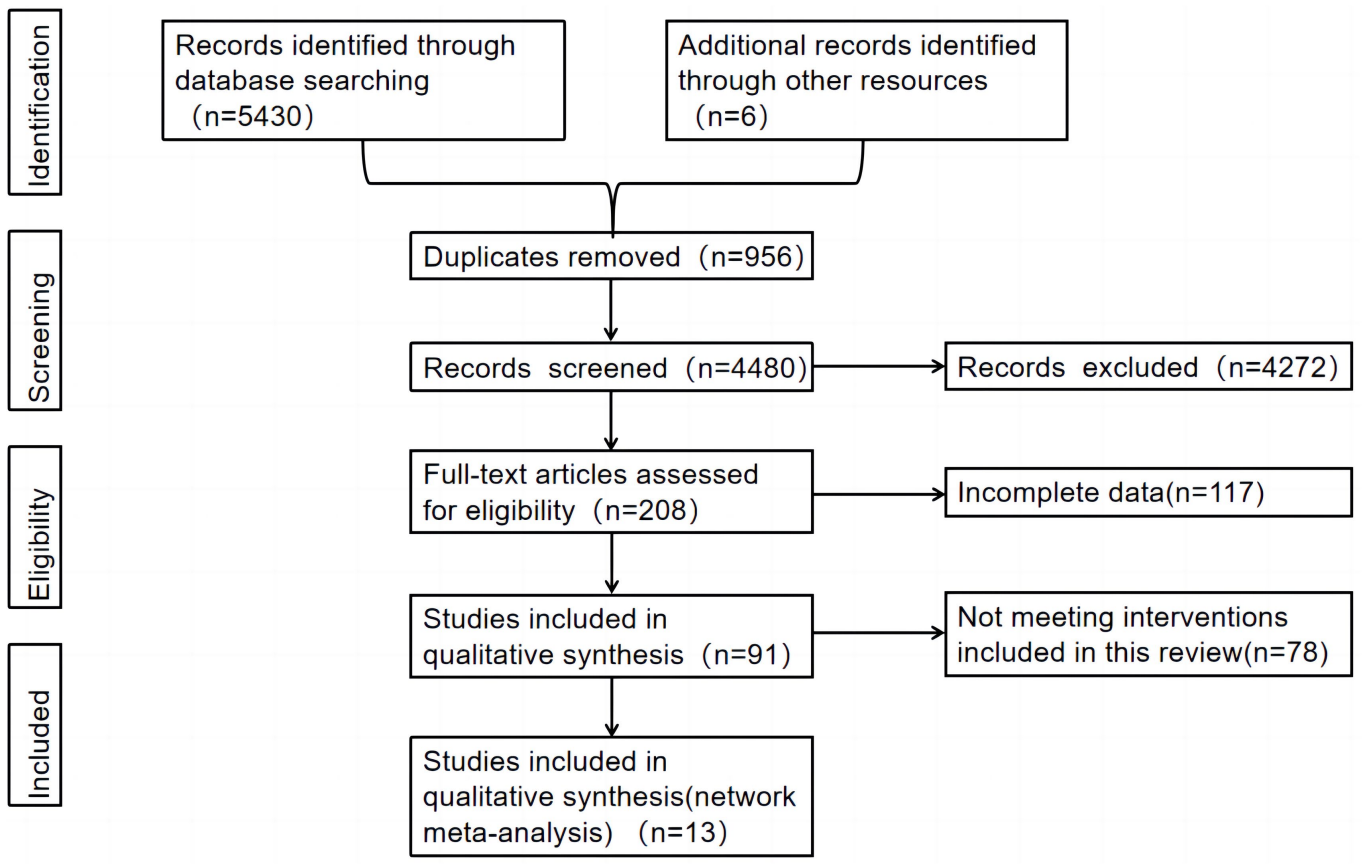
Flow diagram of literature selection.

Hence, 5,436 documents were retrieved from the electronic database and imported into the Endnote. To start with, the research group searched for duplicate literature and 956 duplicate articles were removed. Then, their abstracts were read, and 4,272 articles with inconsistent research topics were excluded. Last but not least, after reading the full text, 117 articles were excluded due to incomplete data, and 78 were excluded because the intervention measures did not meet the inclusion criteria of this article. Finally, 13 articles were hereby included, and the details are shown in Figure 1.

### 3.2. Literature characteristics and quality assessment

The research on the relationship between learning flow and academic achievement included 13 articles, involving 3,253 students. Among the 13 studies included in this paper, 7 were from South Korea, 4 from China, 1 from Israel and 1 from the United States. The research period was 2012–2022 (10 years). The number of research students was 3,253, ranging from 6 years old to 60 years old. The study covered grade 1 of the primary school to grade 4 of the university. Interventions included different media learning methods (2 studies), enterprise online training (1 study), game-based learning (3 studies), online learning (4 studies) and computer science courses (3 studies). Among the 13 articles included in this review, one scored less than or equal to 6, categorizing it as low-quality literature. Conversely, one article scored 9 or above, classifying it as high-quality literature. The remaining 11 articles, with scores ranging from 7 to 8, are considered to be of moderate quality. The assessment of research quality indicates that the studies incorporated into this evaluation exhibit a medium to high degree of rigor. The details are shown in Table 3.

**Table 3:** Characteristics of the studies included in the meta-analysis.

Number	Authors	Country	Age (mean + SD)	Educational Level	Total/male/female	Intervention	NOS
1	Chang et al. (2018)	Taiwan, China	19	Undergraduate	T:53/21/32\u00B0C:50/14/36	Different media learning methods T: Using the DGBL Environment C: Using a non-game based CBL environment	7
2	Joo et al. (2012)	Korea	23–58	Adult Education	248/215/33	Enterprise online training	8
3	Park and Lee (2018)	Korea	20–60	College Student	256/93/163	E-learning in online universities	7
4	Lee et al. (2020)	Korea	University grade 2–4	College Student	147/19/128	Online lectures	8
5	Barzilai and Blau (2014)	Israel	10.10 (1.71)	Primary and Junior High School Students	182/111/71	Scaffold learning based on games	6
6	Lee (2021)	Korea	Grade 1–4	College Student	179/42/137	Online learning	7
7	Park et al. (2022)	Korea	21.45 ( 1.84)	Undergraduate	201/25/176	Synchronous or asynchronous online learning	7
8	Kim and Kim (2018)	Korea	Senior High School Grades 1–3	Senior High School Student	251/150/101	Intelligent learning environment	8
9	Yeh and Lin (2018)	Taiwan, China	Grades 4–6	Pupil	275/140/135	Digital creative game	7
10	Joo et al. (2015)	Korea	33.64	College Student	959	Online Computer Application Course in University Environment	7
11	Liu (2016)	Taiwan, China	20–21	College Student	T:55/44/11\u00B0C:55/46/9	Computer Science Course T: Use educational games C: Use simulation software	8
12	Rachmatullah et al. (2021)	United States	Grades 1 and 9–12	Middle School Student	307/141/144/22	Genetics Learning Based on Digital Games	8
13	Liu et al. (2021)	China	18.5	College Student	35/16/19	Mixed Courses in Computer Science 13 weeks	9

### 3.3. Heterogeneity test and statistical analysis

**Figure 2:**
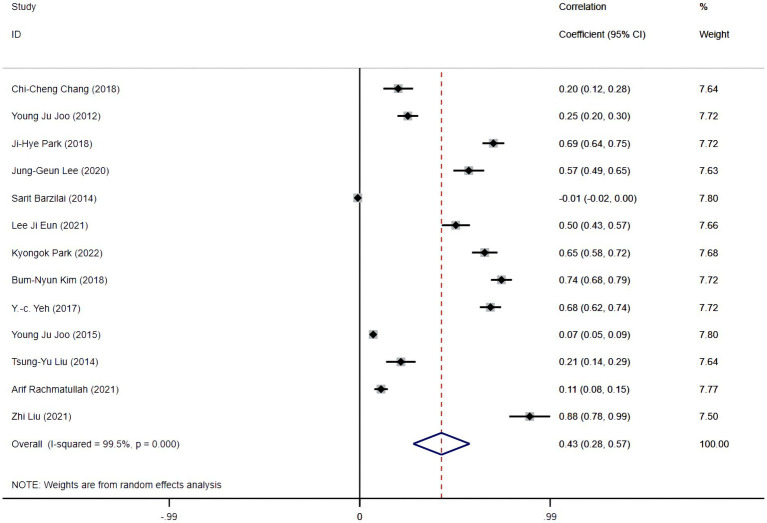
Forest plot on heterogeneity test.

Conversely, the correlation coefficients observed spanned from −0.01 to 0.88. Evaluating these coefficients and their 95% confidence intervals revealed an I-squared heterogeneity of 99.5%, with a significance level of *p* = 0.000, necessitating the adoption of a random effects model for statistical interpretation. Analysis indicated correlation coefficients within confidence intervals of 0.43 (0.28, 0.57), denoting a statistically significant, positive linear association between learning flow and academic performance. The details are shown in Figure 2.

### 3.4. Publication bias and sensitivity analysis

**Figure 3:**
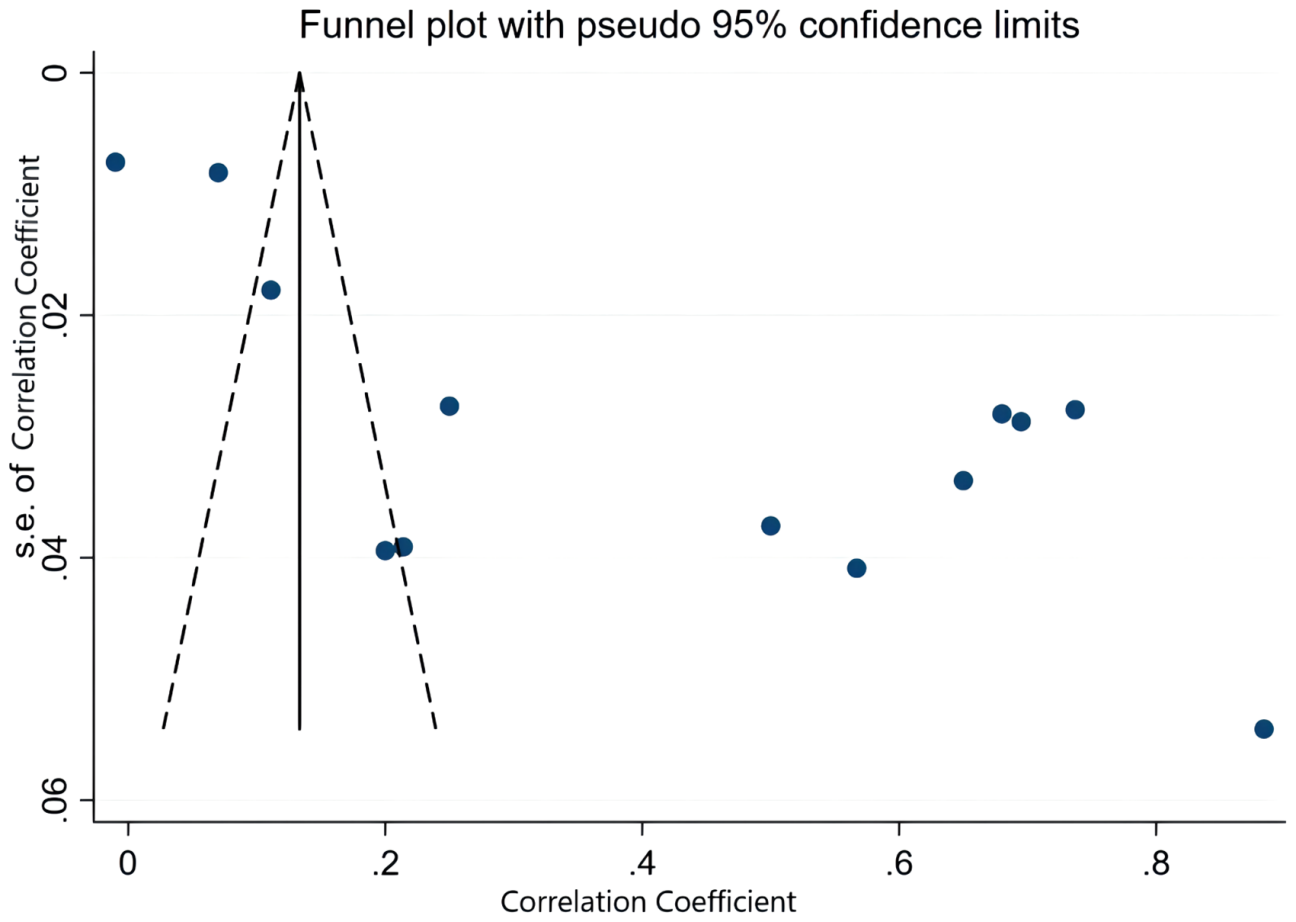
Funnel plot on publication bias.

**Figure 4:**
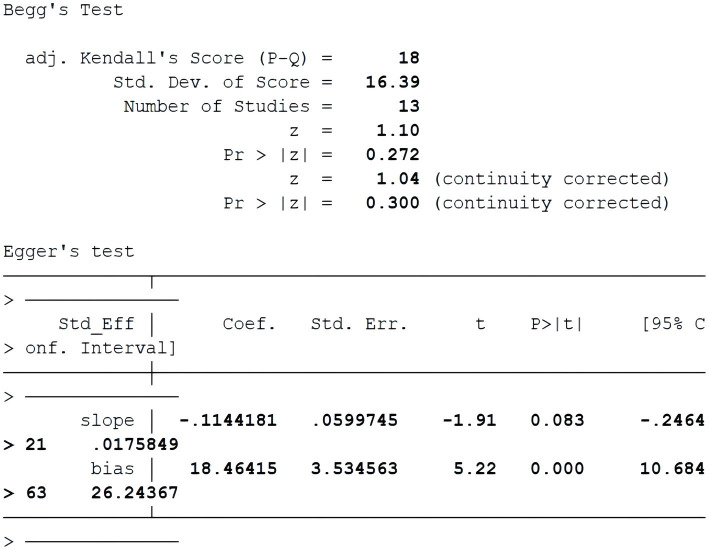
Begg’s test.

**Figure 5:**
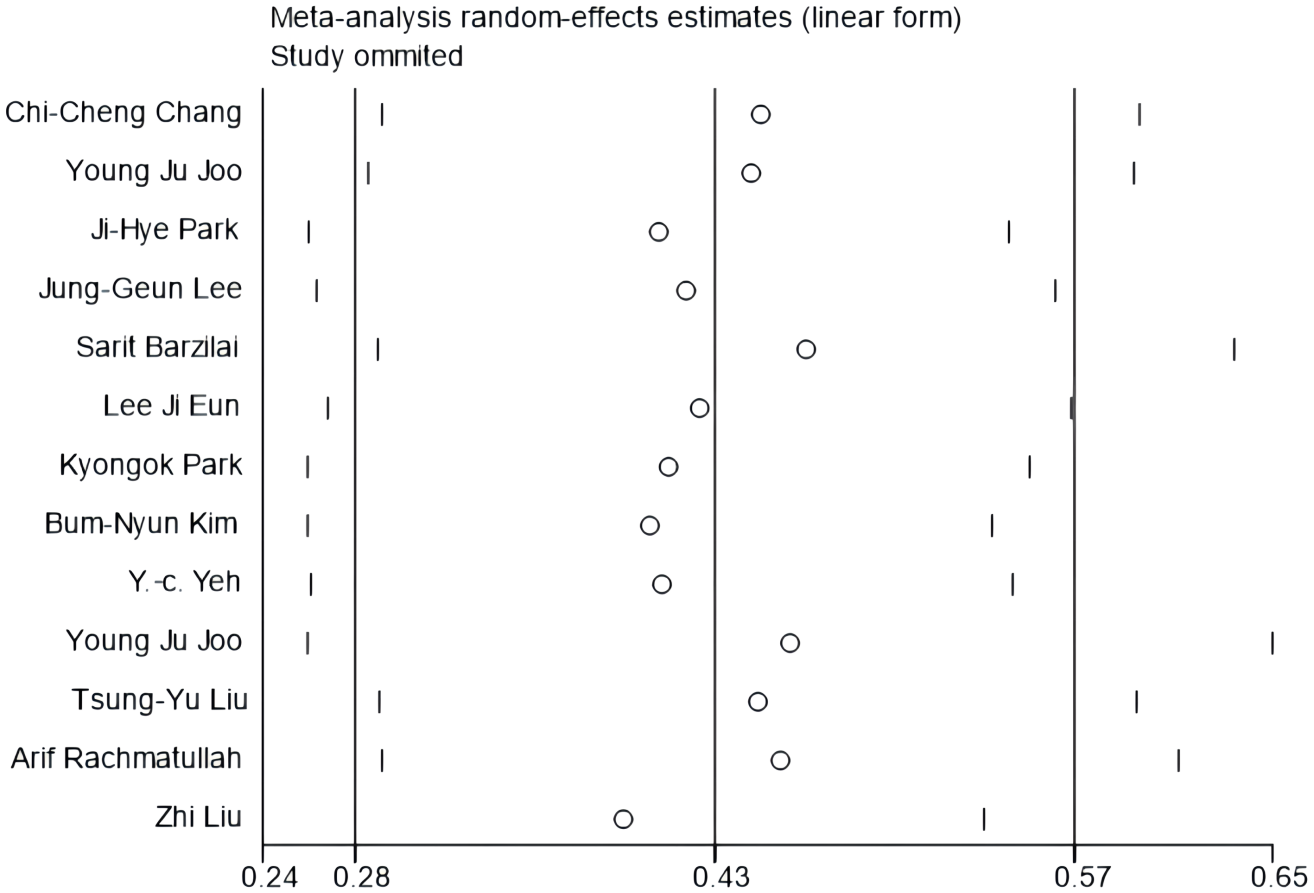
Sensitivity analysis.

To start with, based on the correlation coefficient and its 95% confidence interval, a publication bias test was performed, and a funnel chart was generated, which was considered roughly symmetrical on both sides, indicating no obvious publication bias. The details are shown in Figures 3, 4. We examined the potential publication bias of the correlation coefficient by analyzing its 95% confidence interval and subsequently constructing a funnel plot. The funnel plot illustrates that both the vertical and horizontal axes represent the standard error and effect sizes, respectively, with the confidence interval set at 95%. As points ascend within the plot, they indicate a reduction in standard error, with each circle depicting a separate study (Wijaya et al., 2022). We further scrutinized publication bias through Begg’s and Egger’s tests. Typically, when fewer than 20 studies are included, Egger’s test, with its superior efficiency, becomes more reliable than Begg’s, making Egger’s findings primarily considered. Subsequently, we conducted a meticulous sensitivity analysis, employing a sequential exclusion method (Harris et al., 2021). This approach entailed first removing the initial study and generating a forest plot to observe any consequent alterations in the results. We then reinstated the first paper, proceeded to exclude the second, and developed another forest plot. This process was repeated, sequentially excluding each subsequent study, to determine whether the absence of any specific paper would significantly skew the results. Ultimately, the consistency in outcomes under varying conditions confirmed the stability of our results. The details are shown in Figure 5.

## 4. Discussion

Herein, a total of 5,436 articles were screened, and 13 involving 3,253 students were finally obtained, which revealed a relationship between learning flow and academic achievement.

To begin with, a strong correlation between learning flow and academic achievement was found. Learning flow has been identified as an important influencing factor of academic achievement. Considering the significant role of learning flow when students face the frustration with challenging tasks, low-level learning process makes students unable to complete their studies, which leads to dropout. Learners are immersed in the flow state generated by the current learning experience, so that they can better focus on learning activities and reduce the pressure of learning tasks, thereby having their academic performance improved. This is consistent with our research results. In the state of flow, students have internal motivations, feel controlled and maintain certain concentration, which is the reason that learning immersion has a positive impact on academic performance (Chang et al., 2018). At the same time, our research results reflect this. Learning flow influences academic achievements by modulating personal characteristics, such as self-efficacy, interest, and motivation. When experiencing a state of flow, online learners tend to enjoy the entire online course, pay full attention to the task, actively interact with peers and teachers, and generate self-efficacy in the learning process (Joo et al., 2012). Additionally, learning flow impacts academic performance by shaping curriculum content, particularly the subject knowledge or content structure suitable for students’ age characteristics, current knowledge, and skill levels. According to the educational games in computer courses exploring the relationship between academic performance and learning flow, students claim that using educational games for learning data structures makes them pleasant and excited, and that they become interested in learning when playing educational games with their classmates. Thus, when using similar education games with other subjects, their learning will become more interesting, efficient and effective (Liu, 2016). Last but not least, if the difficulty of the challenge far exceeds the ability, students will feel anxious. If the challenge is too easy to achieve, they will feel bored and disappointed. When challenges and skills reach a balance, learning flow will occur. It demonstrates the learning flow of students as the key factor to their academic success. Generally speaking, higher learning flow means more active participation, more self-discipline in learning habits and better academic performance compared with lower learning flow. Consequently, based on our findings, elevated flow experiences among students can be attributed to positive peer interactions and a deeper comprehension of the course content.

Therefore, some suggestions and references were put forward. Students with high learning flow tend to have better academic performance, making it necessarily important to focus on the learning flow in students’ study. Only by establishing an interesting and supporting learning environment and improving learners’ actual feelings and learning engagement can students’ learning outcomes be improved. Specifically, for online learning or multimedia teaching, it is vital to consider teaching strategies to enhance learners’ flow and improve their achievements when designing and developing courses such as real learning tasks related to promoting higher-order thinking skills, arguments that promote in-depth and constructive discussions among learners, and well-designed multimedia elements that generate greater interest, support efficiency and learning effectiveness. Regarding classroom instruction, it is imperative to define educational objectives clearly. Teachers should simulate authentic dilemmas resonant with students’ experiences, kindling their curiosity, interest, and drive. Course content structuring requires a harmonization of students’ psychological processing and subject logic, necessitating strategic task assignments. During instructional sessions, prioritizing robust teacher-student interaction is paramount. Teachers must monitor individual student engagement, offering tailored guidance and fostering a supportive, encouraging environment. Integrating assessments of the learning journey into overall performance evaluations optimizes the role of learning flow in fostering student growth and enhancing their academic performance.

## 5. Advantages and limitations

In terms of the advantages of this article, on the one hand, it is the first article using the meta-analysis to research the relationship between learning flow and academic performance, while on the other, the literature retrieval and literature screening methods are rigorous and standard, the inclusion and ranking standards are formulated, the data selection is detailed, and the statistical analysis is correct.

However, the present study is also subject to certain limitations. Firstly, the amount of literature is not enough. Secondly, the relationship between learning flow and academic performance may be affected by age and gender, but there are not sufficient relevant data. Thirdly, the relationship between learning flow and academic performance may be affected by countries, and there may be differences in the education system, curriculum and teachers in different countries, which are not taken into account in this paper. Conclusively, this study acknowledges certain limitations and future research directions, including sample size, applicability to broader demographics, and potential biases. Consequently, readers should exercise caution in interpreting the findings. The necessity for broadening the scope of related inquiries is also underscored.

## 6. Conclusion

Our research findings indicate a clear association between learning flow and academic performance. Students with high learning flow levels tend to have better academic performance, but more high-quality literature and larger sample data are still needed to further verify this conclusion. In our research, we propose the following recommendations to teachers and educators striving to enhance student academic performance: Elevate the level of student engagement and flow in the learning process to achieve this objective. This study also prompts a shift in our educational and teaching practices toward a more student-centered approach, placing significant emphasis on student learning experiences.

## Data availability statement

The original contributions presented in the study are included in the article/Supplementary material, further inquiries can be directed to the corresponding author.

## Author contributions

ZJ: Writing – original draft. FQ: Writing – review & editing.
